# Application of Multiparametric Flow Cytometry Panels to Study Lymphocyte Subpopulations in Tuberculin-Positive Cattle

**DOI:** 10.3390/vetsci10030197

**Published:** 2023-03-05

**Authors:** Anabelle Manzo-Sandoval, Laura Jaramillo-Meza, Roxana Olguín-Alor, Luvia Enid Sánchez-Torres, Fernando Díaz-Otero

**Affiliations:** 1 Laboratorio de Inmunología, Centro Nacional de Investigación Disciplinaria en Salud Animal e Inocuidad, Instituto de Investigaciones Forestales, Agrícolas y Pecuarias, Carretera Federal México-Toluca Km 15.5, Ciudad de México 05110, Mexico; 2 Laboratorio Nacional de Citometría de Flujo, Instituto de Investigaciones Biomédicas, Universidad Autónoma de México, Circuito Escolar 33, Ciudad de México 04510, Mexico; 3 Departamento de Inmunología, Escuela Nacional de Ciencias Biológicas, Instituto Politécnico Nacional, Prolongación de Carpio y Plan de Ayala, s/n, Ciudad de México 11340, Mexico

**Keywords:** flow cytometry, multiparametric panel, bovine, lymphocytes, monoclonal antibodies, *Mycobacterium bovis*

## Abstract

**Simple Summary:**

Flow cytometry is a technique that allows identifying different cell populations based on their morphological characteristics and the presence of proteins in the membrane or inside the cell. It is widely used in biomedical research and clinical diagnosis; however, in the veterinary area, its use has been limited due to the reduced availability of fluorochrome-conjugated antibodies that recognize specific proteins. In order to implement multiparametric analyses in the study of bovine tuberculosis, this work designed two panels composed of different conjugates and tested them to identify helper, cytotoxic, activated, and memory T cells in heifers positively and negatively tested for tuberculin (diagnostic test for bovine tuberculosis). Both panels allowed the identification of the cells of interest; in addition, a higher number of activated and memory cells were observed in tuberculin-positive heifers. These panels can also be used in immunopathogenesis studies, as well as in the evaluation of vaccines in cattle.

**Abstract:**

Flow cytometry (FC) is widely used in microbiology, immunology, hematology, and oncology. In the veterinary field, FC enabled the study of the immune response in cattle infected with different pathogens, as well as vaccine testing. However, few fluorochrome-conjugated antibodies recognize bovine antigens, limiting the possible benefits of FC and the implementation of multiparametric analysis for more complex studies. Two cytometry panels with five colors each were designed and implemented for the study and identification of populations and subpopulations of T cells derived from the peripheral blood mononuclear cells of dairy heifers. Both panels detected differences in T cell subpopulations between heifers positively and negatively tested for tuberculin; they detected overexpression of CD25^+^ and CD45RO^+^ in tuberculin-positive heifers after stimulation with a culture filtrate protein extract (CFPE) from *Mycobacterium bovis* (*M. bovis*). We identified subpopulations of T cells from peripheral blood mononuclear cells using two multicolor panels. These panels could be used to analyze total bovine blood in immunopathogenic studies and vaccine development. The same strategy could be implemented in other species of veterinary interest.

## 1. Introduction

Flow cytometry (FC) has been used to quickly examine cell populations in suspension. This technique has contributed importantly to the understanding of the animal immune system [1], providing information on cell size and complexity (relative granularity) [2]. In addition, FC measures fluorescence, which can be emitted by the cells’ own molecules, by fluorescent dyes that stain them, by antibodies conjugated to fluorochromes, or by the transfection and expression of fluorescent proteins [3]. Therefore, it allows the evaluation of other parameters, such as molecular expression, cell viability, and ion mobilization.

In the 1980s, only three fluorochromes could be detected in the same stained sample; and in the 1990s, the detection increased to 11 colors [4,5,6]. Recently, a panel with 28 colors was designed to characterize human myeloid and B cells [7]. One of the advantages of multiparametric panels is that many cell markers are analyzed simultaneously, enabling a more complete and detailed analysis of the populations under study and reducing the required sample volume. An important limitation in the veterinary field is the scarce availability of antibody-fluorochrome conjugates in the market. Moreover, most of these antibodies are labeled with fluorescein isothiocyanate (FITC) or phycoerythrin (PE), which only allows the analysis of two markers at a time. This condition results in a poor characterization of the different cell populations in the sample and a longer processing time, since more samples per individual need to be handled to identify the cell markers of interest [8]. In cattle, FC was used for counting total leukocytes in a blood sample [9], in the study of neutrophil adhesion receptors (CD11/CD18) [10,11], in the analysis of the immune response to *M. bovis* [12,13,14,15] and other pathogens [16,17], in the evaluation of vaccine effectiveness [18,19,20], and in the diagnosis of theileriosis [21]. However, all these panels used a limited number of fluorochromes. On the other hand, few studies focused on characterizing subpopulations of peripheral blood T lymphocytes from cattle positive and negative to the tuberculin test, a diagnostic test for bovine tuberculosis. Bovine tuberculosis (bTB) is an animal and zoonotic disease that causes significant financial loss worldwide and represents a public health hazard. The causative agent of bTB is *Mycobacterium bovis*, a member of the *Mycobacterium tuberculosis* complex [22]. Studying the dynamics of lymphocyte subsets in the circulation of cattle infected experimentally with *M. bovis* revealed a sequential involvement of γδ cells, then CD4 cells, and later in the infection, CD8 cells. CD4 cells appeared to be the most dominant cell population producing interferon-gamma (IFN-γ) and leading to activation of macrophage anti-mycobacterial capabilities, with CD8 cells having a greater involvement in the lysis of infected cells. γδ cells are also a potential source of IFN-γ, although they release lower levels in comparison to CD4 cells [23]. CD25, or surface interleukin 2 receptor (IL2R), is an early marker of lymphocyte activation and a prerequisite for lymphocyte proliferation. On the other hand, CD45RO is a conventional memory marker used extensively to detect memory T cells in humans and cattle [24]. 

This work aimed to design two multicolor panels to analyze different T lymphocyte markers in a single sample by FC and apply them to study lymphocyte subpopulations in tuberculin-positive and -negative cattle, in field conditions.

## 2. Materials and Methods

### 2.1. Animals and Experimental Design

Ten 1–1.5-year-old Holstein-Friesian heifers were selected from a dairy herd located in the state of Hidalgo, Mexico. Five heifers were positive for bovine tuberculosis, and five were negative, as determined by the comparative cervical tuberculin test and IFN-γ assay. Heparinized blood samples were obtained from all animals. The protocol was authorized by the National Center for Disciplinary Investigation in Animal Health and Safety (Centro Nacional de Investigación Disciplinaria en Salud Animal e Inocuidad, CENID-SAI).

### 2.2. Culture Filtrate Protein Extract of M. bovis AN5

The *M. bovis* strain AN5 was grown on Stonebrink medium for 3–4 weeks at 37 °C. Then, the medium was changed, and a small sample collected with a cell spreader was inoculated in Dorset-Henley medium and incubated for 6–8 weeks until a thick bacterial mass (pellicle) formed on the surface. The pellicle was separated by filtration through a wire mesh. The remaining liquid was passed through a Whatman filter paper. Finally, nitrocellulose filters with pore sizes of 1.2, 0.8, 0.45, and 0.22 µm were used to obtain a bacteria-free filtrate defined as the culture filtrate protein extract (CFPE) [25]. The CFPE was used as an antigen to stimulate peripheral blood mononuclear cells (PBMCs) because it consisted of a protein complex including mycobacterial proteins such as MBP70, MBP83, MBP64, and MPT51 [26].

### 2.3. Isolation and Culture of PBMCs

PBMCs were isolated from heparinized bovine blood by density gradient using Ficoll-Paque ^TM^ PLUS (GE Healthcare, Uppsala, Sweden). Once purified, PBMCs were seeded in RPMI 1640 culture medium with 2 mM L-glutamine, 25 mM HEPES, 5 × 10^−5^ M β-mercaptoethanol, 100 U/mL of penicillin, 100 μg/mL of streptomycin, 2 g/L of sodium bicarbonate, and 10% bovine fetal serum (Sigma-Aldrich, St. Louis, MI, USA, EE. UU.). PBMCs were seeded into 24-well plate at a concentration of 1 × 10^6^ cells/mL and stimulated with 0.3 µg/µL CFPE. Untreated cells (control) were included. Then, PBMCs were cultivated a 37 °C in a 5% CO_2_ atmosphere for three and nine days.

### 2.4. Monoclonal Antibodies

The antibodies used in this study are listed in Table 1. Two panels were designed to identify leukocytes (CD45^+^), T lymphocytes (CD3^+^), helper T lymphocytes (CD4^+^), cytotoxic T lymphocytes (CD8^+^), activated T lymphocytes (CD25^+^), and memory cells (CD45RO^+^).

### 2.5. Staining for FC

Pre-staining, trypan blue was used to identify dead cells. Cell viability was 96% on day 3 and 90% on day 9. For each panel, a single tube with stimulated cells and another tube with non-stimulated cells from each animal were stained.

Three-day PBMCs cultures were stained with Panel 1 using monoclonal antibodies (mAbs) that recognized the bovine antigens CD45, CD4, CD8, and CD25. An anti-human CD3 mAb cross-reactive with bovine was included.

PBMCs cultures incubated for nine days were stained with Panel 2, which included anti-CD3, CD4, CD8, CD25, and CD45RO mAbs. All antibodies were pre-titrated to determine optimal working concentrations.

For both panels, 1 × 10^6^ cells were placed in microtubes and centrifuged at 400× *g* to remove the culture medium. Then, cells were washed with 200 µL of wash buffer (phosphate-buffered saline and 1% bovine serum albumin) (Sigma-Aldrich) and centrifuged at low speed; supernatant with cell debris was eliminated. Subsequently, cells were resuspended in 200 µL of blocking buffer (phosphate-buffered saline and 20% bovine serum) and incubated for 15 min. This step also was included for each panel before adding the secondary antibodies to avoid non-specific binding. PBMCs were washed with 200 µL of wash buffer and centrifuged at 400× *g*. The entire process was performed at 4 °C.

In Panel 1, which used intracellular anti-human CD3 mAb, the cells were first stained with antibodies against surface antigens (CD45, CD4, CD8, and CD25), washed with wash buffer and centrifuged at 400× *g*. Then, PBMCs were incubated with anti-IgG2a-PerCP antibody, washed with wash buffer and centrifuged at 400× *g*. Cells were permeabilized with Fix/Perm Buffer (BioLegend), washed with Perm Wash Buffer (BioLegend), and stained with anti- human CD3 mAb, following the manufacturer’s instructions. In Panel 2, cells were stained with anti-bovine CD45RO, washed with wash buffer and centrifuged at 400× *g*. After, secondary antibody anti-mouse-IgG1-FITC was added. Subsequently, PBMCs were washed with wash buffer and centrifuged at 400× *g*. Anti-bovine CD3, CD4, CD8, and CD25 antibodies were added, and PBMCs were washed and centrifuged again. Cells were incubated with the secondary antibodies (anti-mouse-IgG1-BV and anti-mouse-IgG2a-PerCP). After incubation, the cells were washed, centrifuged, and fixed with 4% paraformaldehyde (Sigma-Aldrich) for 10 min. PBMCs were washed and resuspended in wash buffer. Finally, cells were stored 12 h at 4 °C before flow cytometry analysis. Each staining was carried out in 50 µL of staining buffer (antibodies + phosphate-buffered saline and 1% bovine serum albumin). All incubations lasted 15 min, and the whole process was performed at 4 °C.

**Figure 1:**
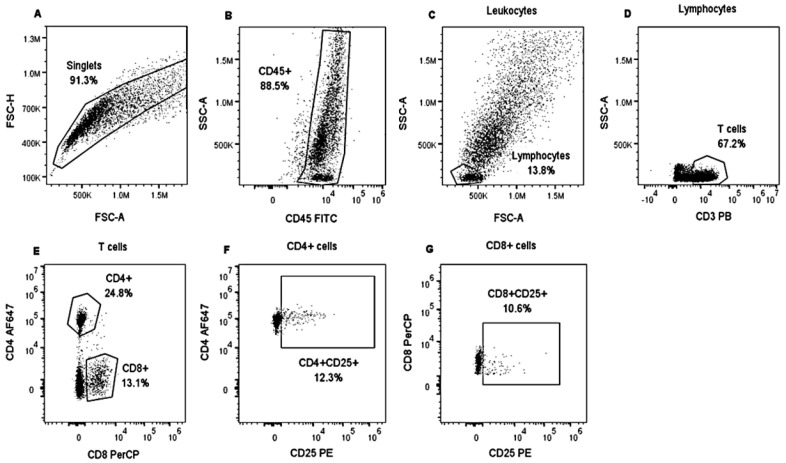
Analysis strategy to identify lymphocyte populations in bovine PBMCs. PBMCs from heifers positively and negatively tested for tuberculin were stimulated with CFPE and cultivated for three days. An unstimulated control was also included. Then, PBMCs were stained with Panel 1. Singlets were gated on FSC-H and FSC-A (**A**). Then, SSC gating was applied, and CD45^+^ expression was analyzed to visualize leukocytes (**B**). Lymphocytes were identified based on SSC and FSC properties (**C**). T lymphocytes were identified by assessing CD3^+^ expression (**D**). T cells were separated into two groups based on CD8^+^ and CD4^+^ expression (**E**), and CD25^+^ expression was analyzed in both T cell populations (**F**,**G**). PBMCs from a tuberculin-negative heifer stimulated with CFPE.

**Figure 2:**
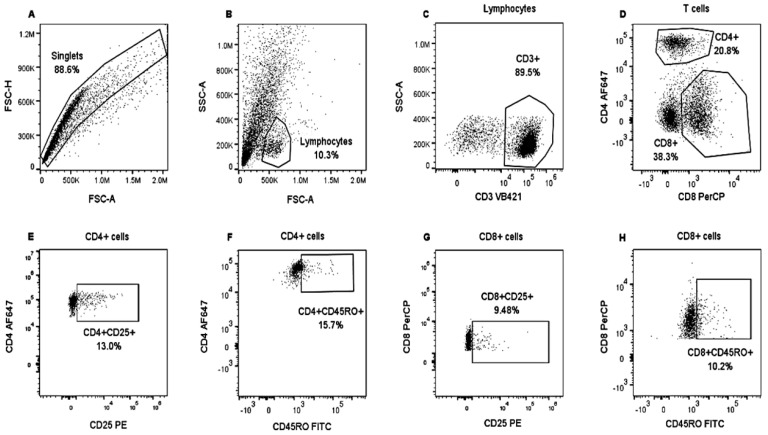
Analysis strategy to identify lymphocyte populations in bovine PBMCs. PBMCs from heifers positively and negatively tested for tuberculin were stimulated with CFPE and cultivated for nine days. An unstimulated control was also included. Then, PBMCs were stained with Panel 2. Singlets were gated on FSC-H and FSC-A (**A**). Lymphocytes were identified from the singlets based on SSC and FSC properties (**B**). T lymphocytes were identified by applying SSC and evaluating CD3^+^ expression (**C**); these were separated into CD8^+^ and CD4^+^ T cells (**D**). CD25^+^ (**E**,**G**) and CD45RO^+^ (**F**,**H**) expression were analyzed in both T cell populations. PBSCs from a tuberculin-negative heifer and stimulated with CFPE.

Each panel included a non-stained control, compensation controls (CC), and Fluorescence Minus One (FMO) controls. FMO were used for the correct quadrant positions for CD25 and CD45RO molecules. The CytoFLEX “S” flow cytometer (Beckman Coulter) was used, which was equipped with a set of four lasers and filters: UV laser (375 nm): 450/40, 660/20; violet laser (405 nm): 450/50, 525/50, 610/20; blue laser (488 nm): 525/40, 585/42, 610/20, 690/50, 780/60; red laser (638 nm): 660/20, 712/25, 780/60 were used to collect at least 10,000 events in singlets gate. Data were analyzed using FlowJo software v.10.8.1 (Becton Dickinson). Cells were gated on forward-scatter height (FSC-H) vs. forward-scatter area (FSC-A) to exclude doublets. For each lymphocyte subset, we applied a specific gating strategy (Figure 1 and Figure 2).

### 2.6. Statistical Analysis

Statistical analyses were performed using Sigma Stat v3.5 software. Differences among the two groups and controls were evaluated by Kruskal–Wallis one-way ANOVA. The Mann–Whitney analysis of variance by rank was used to evaluate the differences between 3-day and 9-day cultures. Both statistical tests allowed to assess whether the samples followed a normal distribution. Asterisks indicate statistical significance. * *p* < 0.05; ** *p* < 0.01; *** *p* < 0.001.

## 3. Results

### Lymphocyte Subpopulations in Tuberculin-Positive and -Negative Animals

**Figure 3:**
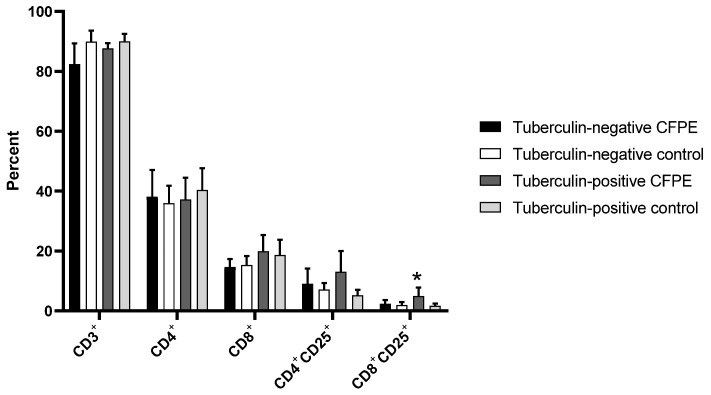
T lymphocyte subpopulations identified with Panel 1 in PBMCs from tuberculin-positive and tuberculin-negative heifers. Cells were cultivated for three days. * *p* < 0.05.

Panel 1, which was used in the 3-day cultures, showed a proportion of T lymphocytes between 82 and 90% in control and CFPE-stimulated cultures of tuberculin-positive and -negative animals. There was no difference in the CD4^+^ population between tuberculin-positive and -negative animals. Tuberculin-positive cattle had higher numbers of CD8^+^ and CD4^+^CD25^+^ cells after stimulation with CFPE, although this was not significant. However, there was a significant increase in CD8^+^CD25^+^ cells from CFPE-stimulated tuberculin-positive heifers compared to the unstimulated control (*p* < 0.05) (Figure 3).

**Figure 4:**
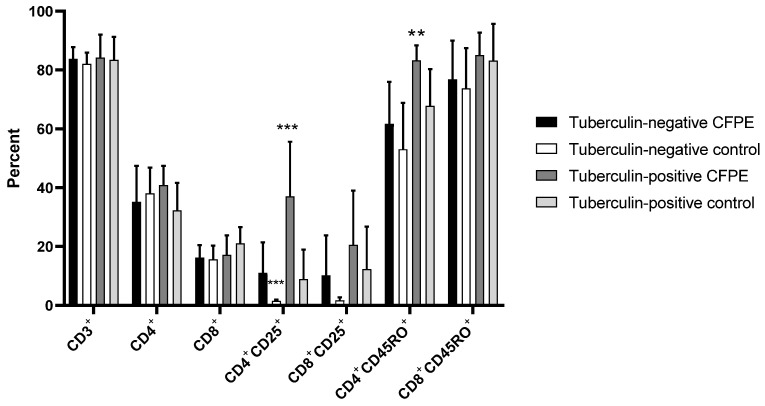
T lymphocyte subpopulations identified with Panel 2 in PBMCs from tuberculin-positive and tuberculin-negative heifers. The cells were cultivated for nine days. ** *p* < 0.01; *** *p* < 0.001.

Panel 2, which was used in 9-day cultures, showed a proportion of 82–84% CD3^+^ cells in tuberculin-positive and -negative animals, both in control and CFPE-stimulated cultures. There was no difference in CD4^+^, CD8^+^, CD8^+^CD25^+^, and CD8^+^CD45RO^+^ populations between groups. CFPE-stimulated PBMCs from the tuberculin-positive group showed a significantly higher proportion of CD4^+^CD25^+^ cells (37%) than the unstimulated control and the tuberculin-negative groups (both stimulated and unstimulated) (*p* < 0.001). Regarding CD4^+^CD45RO^+^ memory cells, there was a significant difference (*p* < 0.01) between the stimulated tuberculin-positive group (85%) and the tuberculin-negative control animals (74%) (Figure 4).

The percentages of lymphocyte subpopulations at 3 and 9 days of culture were also different; activated CD4^+^CD25^+^ cells decreased significantly (*p* < 0.001) in control tuberculin-negative animals. Conversely, in the stimulated cells of tuberculin-positive animals, an increase in CD4^+^CD25^+^ cells (37.1%) was observed at the end of the experiment (*p* < 0.05).

The CD3^+^CD4^−^CD8^−^ cell population was found in both panels in a high percentage (40% approximately). Due to the phenotype and the proportion in which they were found, these could have been γδT cells. Since our study did not contemplate these cells initially and there was no specific antibody for their identification, this subset was not analyzed further.

## 4. Discussion

One of the main applications of FC is immunophenotyping or identifying cell populations and subpopulations based on the specific intracellular or surface expression of molecules. In this work, we identified T-cell subpopulations within PBMCs of 1–1.5 years-old heifers by applying two staining panels of five fluorochromes, each using commercial antibodies.

Selecting different fluorochromes was essential for the design of multicolor panels. Unfortunately, most commercial mAbs that recognize bovine antigens were not conjugated to fluorochromes or were only conjugated to FITC, PE, or AF647. In order to broaden a panel, the most common strategy is to use conjugated secondary antibodies [27] or cross-species-reactive antibodies, specifically those that recognize human differentiation groups and cross-react with other animal species [28,29,30].

The anti-bovine antibodies used belonged to IgG1 and IgG2a subclasses, some of them conjugated to fluorochromes; however, in other cases, secondary antibodies were required. This happened with the unconjugated anti-bovine CD8 antibody, which was detected by incorporating an anti-mouse IgG2a-PerCP because, in this panel, the anti-bovine CD4-AF647 antibody and also the IgG2a subclass was used, there was a risk that the secondary antibody would bind to both IgG2a antibodies. When cells were acquired on the cytometer, CD4^+^ and CD8^+^ populations were defined, and no double positive cells were observed, indicating that there was no interaction or reactivity of this secondary antibody with the primary conjugated antibody, and that this reactivity was directed only to the unconjugated primary antibody of the same subclass.

In Panel 2, including the above-mentioned antibodies, three IgG1 subclasses (unconjugated anti-CD3, anti-CD25-PE and unconjugated anti-CD45RO) were included. To prevent secondary antibodies (anti-mouse IgG1-BV421 and anti-mouse IgG1-FITC) from binding indistinctly to primary antibodies, staining was initiated by adding unconjugated an anti-CD45RO antibody, and in a second step, the secondary anti-mouse IgG1-FITC antibody. Subsequently, the rest of the conjugated (anti-CD4-AF647, CD25-PE) and non-conjugated (anti-CD3 and anti-CD8) primary antibodies were added, and as a last step, anti-mouse IgG1-BV421 and anti-mouse IgG2a-PerCP secondary antibodies were added, with no evidence of binding between them and the respective subclasses antibodies previously conjugated. We do not have a concrete explanation for this. Initially, we thought that the molecular weight of the fluorophores could influence the interaction between secondary antibodies and conjugated primary antibodies. According to the literature, the molecular weight of AF647 was 1.3 kDa and of PerCP was 35 kDa; while the molecular weights of FITC, PE, and BV421 were 389 Da, 240 kDa and, 60–80 kDa, respectively [31]. As there are significant differences between the molecular weights, we believe that this was not a factor that prevented the binding of secondary antibodies.

On the other hand, it is possible that fluorochromes conjugated to primary antibodies block epitopes that were recognized by secondary antibodies, or that this conjugation caused structural changes that substantially affected the site of interaction between them, so that they would not bind to the conjugated antibody of the same subclass as occurs with unconjugated primary antibodies, which did not undergo any conformational change [32]. It is necessary to include controls to confirm that the secondary antibody only bound to the corresponding unconjugated primary antibody.

About cross-species-reactive antibodies, in Panel 1, we used the anti-human CD3 antibody (clone CD3-12), which was used to identify animal T cells [33,34,35]. This antibody recognized a highly conserved peptide within the cytoplasmic portion of the CD3ε chain [36,37]. The percentage of T lymphocytes detected with the anti-human CD3 antibody in control and stimulated cells of tuberculin-negative and positive heifers was between 82 and 90%, not appreciating a difference between groups.

The proportion of CD4^+^ cells in Holstein heifers up to 6 months of age was reported to be 20–35% [38]. We found this population to be 32–40% in both stimulated and unstimulated cells of tuberculin-positive and -negative cattle, remaining stable at 3 and 9 days. In studies with cattle infected with *M. bovis*, CD4^+^ and γδ TCR^+^ cells were shown to be the predominant cell populations in response to CFPE stimulation [12], but we did not observe an effect of CFPE.

In Holstein Friesian cattle, the CD8^+^ population remained relatively constant (10–15%) throughout the cattle’s lifetime [39]. In this study, the CD8^+^ population in tuberculin-positive heifers was higher than in negative heifers (at 3 and 9 days); however, this difference was not significant.

Regarding activated cells, previous studies showed that PBMCs from 6-month-old crossbreed cattle infected with *M. bovis* and stimulated with bovine PPD after six days in culture had a proportion of 60% of CD4^+^CD25^+^ and 16% of CD8^+^CD25^+^ [40]. In this work, we found a proportion of 37% and 20%, respectively, in positive animals after nine days of culture with CFPE stimulation. Conversely, the proportions after 3 days of CFPE stimulation were 13% for CD4^+^CD25^+^ and 5% for CD8^+^CD25^+^. The differences between the previous study and ours may have been due to the nature of the stimulus, since PPD is a mixture of peptides and CFPE consists of complex proteins. The age and breed of bovines could also influence these differences. The proportion of activated cells in tuberculin-positive animals after stimulation was higher than that of tuberculin-negative animals; CD8^+^CD25^+^ cells were significantly different after both 3 and 9 days of culture. The IL-2 receptor CD25, used as a surface marker, was involved in the activation and regulatory function of lymphocytes, as well as in the division and proliferation of memory T cells. In cattle, different T cell subpopulations were found to be regulated by CD25 in response to the stimulus of mycobacterium antigens [40].

Memory T cells mount rapid immune responses; the conventional memory markers CD45RO and CD45RA are used to detect memory T cells in humans and cattle [41]. CD4^+^CD45RO^+^ and CD8^+^CD45RO^+^ memory cells were measured after nine days of culture; previous reports indicated differences in this population from the sixth day of stimulation. Tuberculin-positive animals showed a proportion of 83% in CD4^+^CD45RO^+^ cells and 85% in CD8^+^CD45RO^+^ cells after stimulation. It was reported that the proportion of CD45RO^+^ cells in crossbred cattle infected with *M. bovis* and stimulated with bovine PPD after six days in culture was 70% for CD4^+^CD45RO^+^ and 40% for CD8^+^CD45RO^+^. Notably, in that study, the cell subpopulation analysis was performed 12 months after the heifers were infected; therefore, the animals were more than one year old. Maue et al. reported that CD4^+^CD45RO^+^ cells represent a subset of effector/memory cells, and that CD45RO^+^ cell subsets are responsible for proliferative recall responses to mycobacterial antigens [40]. Recent studies reported that in PBMCs from healthy cattle (Wye Angus) aged from 1 year to 24 months, activated with a cocktail that contained monensin sodium salt (1.5 mM), phorbol 12-myristate 13-acetate (0.0405 mM), and ionomycin calcium salt (0.67 mM), the proportion of CD45RO^+^ in γδ, CD4+ and CD8+ T cells was 90%, 60%, and 30%, respectively. They propose that the CD45RA/RO distribution pattern on the T cells was associated with distinct T cell subtypes and that it was necessary to identify novel markers for memory T cell populations in cattle [24].

A subset CD3^+^CD4^−^CD8^−^ was evident with Panel 1 and Panel 2, according to the phenotype and percentage, which showed that these could have been γδT cells [42]. γδT cells represent between 15 and 60% of the circulating lymphocytes in the bovine system and are known as innate-like cells [43]. Blood γδT cells are WC1^+^. WC1 (workshop cluster 1) is the γδ cell-specific surface receptor; this molecule functions as a hybrid pattern recognition receptor (PRR). This cell population participates in direct cytotoxicity as well as in antigen presentation [44] and produces IL-17 and IFN-γ to attract neutrophils and activate macrophages [45]. The initial aims of this study did not contemplate identifying these cells. However, considering that γδT cells are key factors in bTB pathology playing a crucial role in granuloma formation [46], further studies should include a deeper characterization of this subset to compare γδT cells in tuberculin-positive and -negative cattle.

The findings presented in this study described differences in CD25 and CD45RO expression between tuberculin-positive and -negative animals. Future research should include other markers such as γδ TCR, CD172a, CD14, MHCII, CD21, CD62L, CD11b, and CD45RA to study the bovine immune system and to identify similarities and differences between animals infected with *M. bovis* and healthy animals, in order to improve diagnostic systems and vaccine evaluation.

## 5. Conclusions

Both multiparametric panels were useful for analyzing lymphocyte populations of heifers; our data indicated that the expression of CD25 and CD45RO was increased in CD4^+^ and CD8^+^ cells from tuberculin-positive cattle. These panels can be applied in research focused on studying the bovine immune system under different scenarios; for example, to determine the efficacy of vaccines of interest for livestock or to follow up cell populations during the course of an infection.

## Figures and Tables

**Table 1:** Monoclonal antibodies and selected fluorochrome used in the current study.

Specificity	Clone	Isotype	Conjugate	Source ^a^
^1^ Bovine CD45	CC1	Mouse, IgG1	FITC	Bio-rad
^1^ Human CD3	CD3-12	Rat, IgG1	Pacific Blue	Bio-rad
^1,2^ Bovine CD4	CC8	Mouse, IgG2a	Alexa Fluor 647	Bio-rad
^1,2^ Bovine CD8	CC63	Mouse, IgG2a	Unconjugated	Bio-rad
^1,2^ Bovine CD25	IL-A111	Mouse, IgG1	RPE	Bio-rad
^2^ Bovine CD3	MM1A	Mouse, IgG1	Unconjugated	VMRD
^2^ Bovine CD45RO	GC42A1	Mouse, IgG1	Unconjugated	VMRD
^1,2^ Mouse IgG2a	344701	Rat, IgG1	PerCP	R&D Systems
^2^ Mouse IgG1	NA	Goat polyclonal IgG	FITC	Jackson ImmunoResearch
^1^ Mouse IgG1	RMG1-1	Rat, IgG	Brilliant Violet 421	BioLegend

FITC, fluorescein isothiocyanate, RPE, R-Phycoerythrin, PerCP, Peridinin-chlorophyll Protein Complex and NA, not applied. ^a^ VMRD, VMRD Inc., Pullman, WA, USA; Bio-Rad, Bio-Rad Inc., Hercules, CA, USA; R&D Systems, R&D Systems Inc., McKinley Place, Minneapolis, MN, USA; Jackson ImmunoResearch, Jackson ImmunoResearch Inc., West Baltimore Pike, West Grove, PA, USA, BioLegend, BioLegend Inc., BioLegend Way, San Diego, CA, USA. ^1^ Panel 1. ^2^ Panel 2.

## Data Availability

The data obtained and analyzed in this study are available from the corresponding author upon request.
